# Supplementary material for: Redox-responsive peptide-based complex coacervates as delivery vehicles with controlled release of proteinous drugs
Source: Commun Chem. 2023 Nov 7;6:243. doi: 10.1038/s42004-023-01044-8 (PMC10630460; doi:10.1038/s42004-023-01044-8)
Supplement: Supplementary file 2 — Supplementary Information [file 42004_2023_1044_MOESM2_ESM.pdf]

*Supplementary information*

# Redox-responsive peptide-based complex coacervates as delivery vehicles with controlled release of proteinous drugs

Jiahua Wang[1,*], Manzar Abbas[2,3], Yu Huang[1,*], Junyou Wang[4] and Yuehua Li[1,*]

[1]Department of Radiology, Shanghai Jiao Tong University Affiliated Sixth People's Hospital, Shanghai, 200233, China.

[2]Department of Chemistry, Khalifa University of Science and Technology, P.O. Box 127788, Abu Dhabi, UAE Abu Dhabi.

[3]Advanced Materials Chemistry Center (AMCC), Khalifa University of Science and Technology, P.O. Box 127788, Abu Dhabi, UAE

[4]State Key Laboratory of Chemical Engineering and Shanghai Key Laboratory of Multiphase Materials Chemical Engineering, East China University of Science and Technology, Shanghai 200237, China

[*] Correspondence: jiahuawang@163.com; yuhuang6y@163.com; liyuehua77@sjtu.edu.cn

**This material includes**

**Supplementary Methods**

**Supplementary figure 1.** Turbidity of metabolites/$R_{10}$ mixtures as a function of metabolites concentration.

**Supplementary figure S2.** Turbidity of metabolites/$R_{10}$ as a function of added NaCl with different concentrations of NADPH or ATP.

**Supplementary figure S3.** Coacervation results were obtained by combining pairs of NADPH/peptide.

**Supplementary figure S4.** Turbidity decay is caused by NADPH/$R_{10}$ droplets coalescence and the turbidity decay in presence of $H_2O_2$ and GSH.

**Supplementary figure S5.** Salt stability of NADPH/peptide coacervates that prepared in PBS buffer.

**Supplementary figure S6.** Turbidity titration of NADPH/DG$R_5$Cs-sC$R_5$GD coacervates by using TCEP and $H_2O_2$ as reducing and oxidizing agents respectively.

**Supplementary figure S7.** Turbidity of NADPH/$R_{10}$ coacervates and encapsulation efficiency of tPA as a function of tPA concentration.

**Supplementary figure S8.** Releasing of FITC-tPA from NADPH/DG$R_5$Cs-sC$R_5$GD coacervates by redox chemistry.

**Supplementary figure S9.** Images of NADPH/R*pep*-RGD coacervate droplets with activated platelets after coalescence of the droplets.

**Supplementary figure S10.** Brightfield and fluorescence images of Hela cells interacted with NADPH/peptide coacervates.

**Supplementary figure S11.** Slowly fusion of NADPH/R*pep*-RGD coacervate droplets.

**Supplementary figure S12.** Brightfield and fluorescence images of platelets interacted with NADPH/$R_{10}$ coacervates.

## Supplementary Methods

### *Materials*

Nicotinamide adenine dinucleotide phosphate (NADPH/$NADP^+$), nicotinamide adenine dinucleotide (NADH), and adenosine triphosphate disodium salt (ATP) were purchased from Roche. Poly-L-lysine hydrobromide (pLys, 15–30 kDa), glutathione (GSH), tris(2-carboxyethyl) phosphine (TCEP), hydrogen peroxide ($H_2O_2$), pyruvate, glucose-6-phosphate (G6P), glucose-6-phosphate dehydrogenase (G6PD), lactic dehydrogenase (LDH), fluorescein isothiocyanate (FITC), 1,1’-dioctadecyl-3,3,3’,3’-tetramethylindocyanine perchlorate (Dil, lipid membrane probe), thrombin, acid citrate dextrose (ACD), sodium chloride and phosphate-buffered saline (PBS) were purchased from Sigma Aldrich and used without further purification. Peptides $R_{10}$, $K_{10}$, and $K_{20}$, were purchased from Alamanda Polymers. $R_3C$, $R_4C$, $DGR_5C$, $R_5C$, $(RG)_5C$, $(RGG)_5C$ were purchased from ScierBio-Tech. Tissue plasminogen activator (tPA) was a product of Boehringer Ingelheim (Germany).

### *Coacervate formation*

Stock solutions were made in Milli-Q water. Samples for turbidity measurements were prepared directly into 96-well plates, by adding, respectively, Milli-Q water or PBS buffer, peptides, and NADPH/$NADP^+$ to a total volume of 100 μL. Mixing was done by gentle pipetting (3×) before each measurement. Samples for the microscopy experiments were prepared in microcentrifuge tubes. A 20 μL aliquot was immediately taken for imaging on a glass slide.

### *Turbidity measurements*

Turbidity measurements were performed in triplicate using a Spark M10 (Tecan) microplate reader. The temperature was kept constant at 25 ± 1°C. The absorbance was measured at 520 nm, where

none of the mixture components absorbed significantly. The absorbance of a well filled with the same volume of water was used as a blank. Samples were shaken for 5 s before every readout. Turbidity (%) was calculated as 100 ($1 - 10^{-Abs}$).

***Preparation of FITC-labelled tPA***

1 mL of FITC solution in DMSO (1.0 mg $mL^{-1}$) was added dropwise into 2 mL of tPA solution in PBS buffer at pH 8.0 (1.0 mg $mL^{-1}$) and stirred at 4 °C in the dark overnight. The mixture was dialyzed against pH 7.4 PBS solution for 2 days (MWCO = 3500 Da), after freeze-drying the purified product FITC-tPA was stored at 4 °C before use.

***Partitioning of labeled tPA***

Localization of labeled FITC-tPA was studied using fluorescence microscopy. ZEISS Axio Vert.A1 fluorescence microscope equipped with 100 × magnification objective was used. Partition efficiency was determined by comparing the ratio of fluorescence intensity in the condensed phase to fluorescence intensity in the outer phase.

***Determination of NADPH and tPA partition coefficients***

In a typical procedure, we began by centrifuging the coacervates dispersion in an Eppendorf tube at 3000x g until the dilute phase was transparent under microscope. After the centrifugation, the coacervate phase had settled at the bottom of the tube. The concentrations of the NADPH or tPA in the supernatant were quantitatively analyzed using Uv-visible spectrophotometry at wavelength of 340 nm or 595 nm with the Bradford assay. Next, we precisely measured the volume of the top (dilute) solution and calculated the NADPH or tPA concentration of the top solution from the standard curve. Subsequently, we calculated the volume and the moles of NADPH or tPA present in

the coacervate phase using the data from the total feed. Then the NADPH or tPA concentration inside the coacervate phase can be obtained. The ratio of NADPH or tPA concentration in the condensed and the diluted phase (partitioning constant) was calculated using the following formula: $K_p = C_c/C_d$, with $C_c$ representing the concentration in condensed phase and $C_d$ representing the concentration in diluted phase.

***Animal Experiments***

All animal experimental procedures were performed in agreement with the guidelines and ethical approval was obtained from the Animal Research Committee of Shanghai Jiao Tong University Affiliated Sixth People's Hospital (approval number: 2022-0188). Healthy male SD rats (≈220 g) were purchased from Shanghai SLAC Laboratory Animal Co., Ltd.

***Isolation of platelets***

Platelets were isolated from whole mouse blood with anti-coagulant acid citrate dextrose (ACD) [blood/ACD = 9/1.5 (v/v)] by differential centrifugation. The blood was placed into a 2 mL tube and centrifuged at 250 × g for 15 min at 4 °C. Platelet-rich plasma (PRP) was obtained by removing red blood cells, and platelets were then collected by centrifugation of PRP at 1200 × g for 5 min. Platelets were re-suspended in PBS buffer with the presence of anti-coagulant ACD at pH 7.4 and counted for experimental use. Activated platelets were prepared by incubation 2 mL inactivated (resting) platelets ($1.0 \times 10^8$ $mL^{-1}$) with 100 µL thrombin (1U $ml^{-1}$) for at least 20 min.

***In vitro thrombolysis in a halo human blood clot model***

Whole mouse blood was collected from the healthy mouse in ACD [blood/ACD = 9/1.5 (v/v)]. Clotting mixture of 5 mL of buffer containing 66 mM tris-HCl, 130 mM NaCl, 45 mM $CaCl_2$, and

10 μL of 500 U $mL^{-1}$ thrombin (pH 7.4). In 96-well plates, one drop of 5 μL of the clotting mixture was placed on the edge of the well bottom, and then another drop of 15 μL of whole blood was added on the opposite edge of the well bottom. Clotting was initiated by mixing the two drops to form a homogenous halo-shaped blood clot around the good edge, leaving the center area empty. The plate was sealed and incubated at 37°C for 30 min for blood clot formation. 80 μL of tPA-NADPH/$R_5Cs$-$sCR_5$ (tPA-NADPH/R*pep*), tPA-NADPH/$DGR_5Cs$-$sCR_5GD$ (tPA-NADPH/R*pep*-RGD), or free tPA (equivalent tPA dose of 0.1 mg $mL^{-1}$) was added into the wells containing halo blood clots at the same time. The dissolution of halo blood clots was determined by measuring absorbance at 510 nm using a plate reader with 5 s orbital shaking, resulting from red blood cells progressively covering the center of the well after clot lysis at 37°C. A negative control was obtained from the addition of 80 μL of PBS (without tPA) only to the halo clot, while a well containing 15 μL of whole blood (no halo clots) and 85 μL of PBS was used as a positive control. The percentage of clot dissolution was calculated by the following equation:

$$\%\text{Clot lysis}=\frac{A_s\text{-}A_n}{A_p\text{-}A_n}\times 100$$

where $A_s$ is the absorbance of the sample well after treatment, $A_n$ is the absorbance of the negative control well, and $A_P$ is the absorbance of the positive control well. Replicates were obtained with blood clots made from the blood of three different donors.

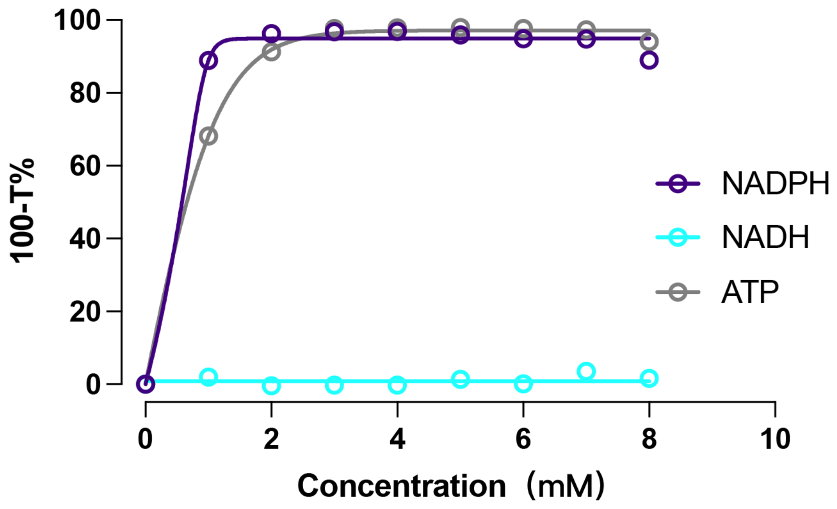


**Supplementary figure 1.** Turbidity of metabolites/$R_{10}$ mixtures as a function of metabolites concentration. The mixtures contained a fixed concentration of 10 mM $R_{10}$ (monomer units).

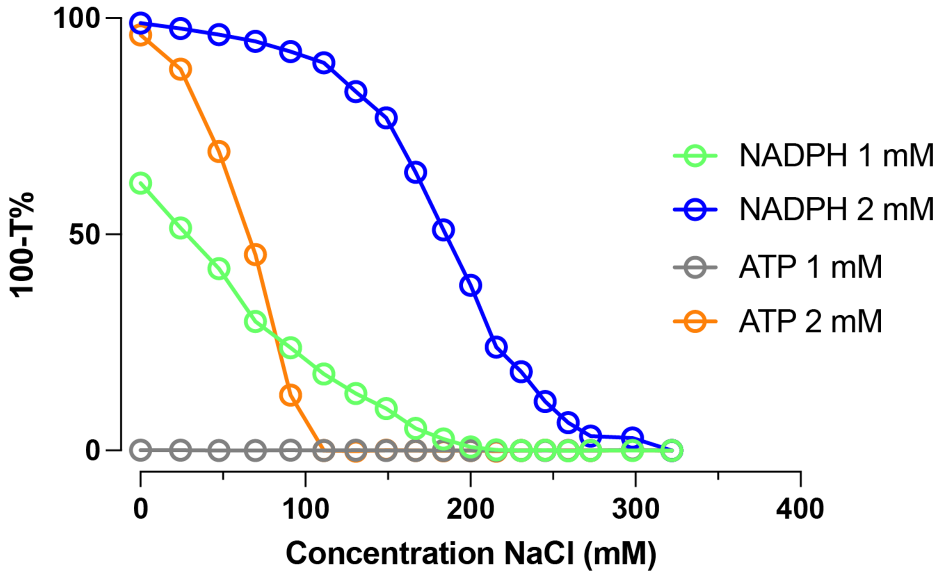


**Supplementary figure 2.** Turbidity of metabolites/$R_{10}$ as a function of added NaCl in different concentrations of NADPH or ATP. The mixtures contained a fixed concentration of 10 mM $R_{10}$ (monomer units, 200 mM stock solution, 10 µL in 200 µL mixture solution).

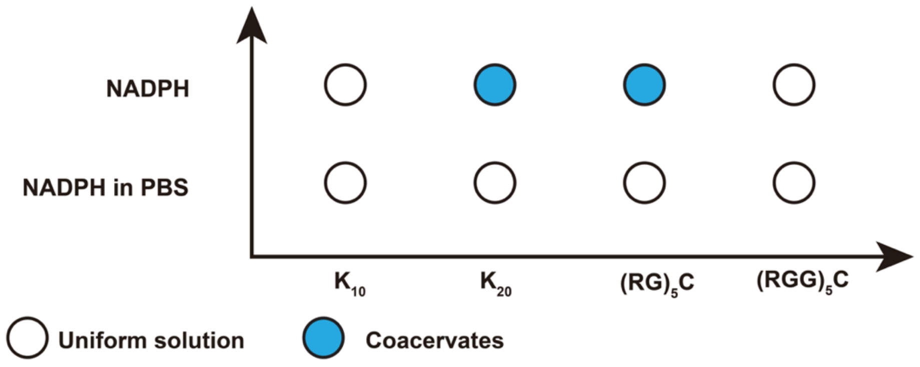


**Supplementary figure 3.** NADPH 4 mM was mixed with cationic peptides at 10 mM (Lysine or Arginine monomer basis). Symbols indicate the observation of uniform solutions (white circles) or coacervates (blue circles).

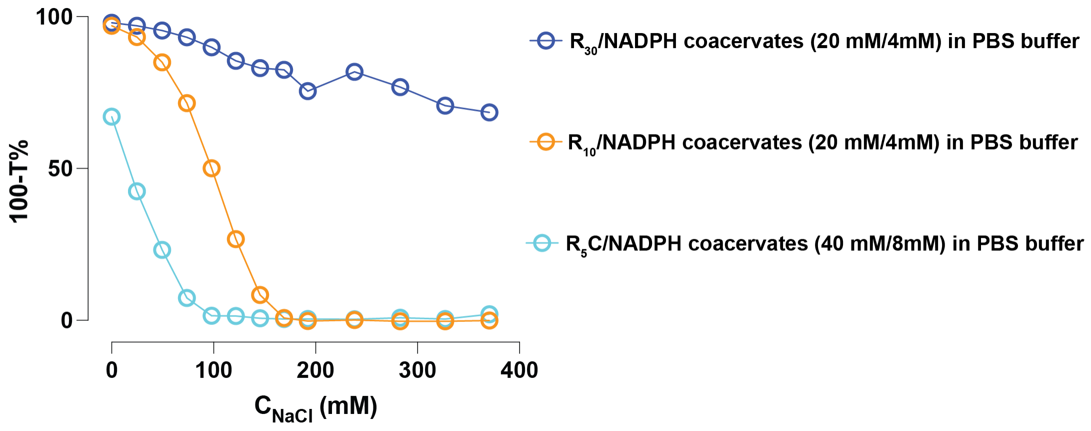


**Supplementary figure 4.** Turbidity of NADPH/peptides mixture as a function of added NaCl with different lengths of arginine-rich peptides. The mixtures were prepared in PBS buffer.

**Coalescence of NADPH/$R_{10}$ coacervate droplets.** It is inevitable that the coacervate droplets in our system coalesce, causing the turbidity to decrease slowly in time. In order to show that GSH and $H_2O_2$ cannot induce the dissolution of coacervates, we conducted a control experiment where NADPH/$R_{10}$ coacervates were prepared in the same conditions, and in the presence of $H_2O_2$ and GSH. The decrease in turbidity is similar to the decrease observed without the addition of $H_2O_2$ and GSH.

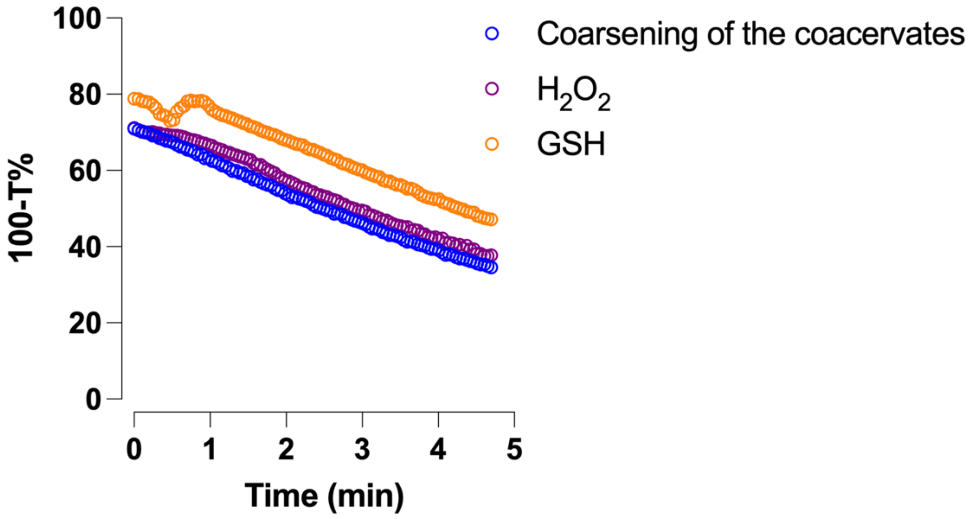


**Supplementary figure 5.** Turbidity decay caused by NADPH/$R_{10}$ droplets coalescence (blue circles), and time-dependent turbidity of NADPH/$R_{10}$ mixtures in presence of $H_2O_2$ (purple circles, 300 mM), and GSH (orange circles, 3 mM). The mixtures contained a fixed concentration of 3 mM NADPH and 20 mM $R_{10}$ (monomer units).

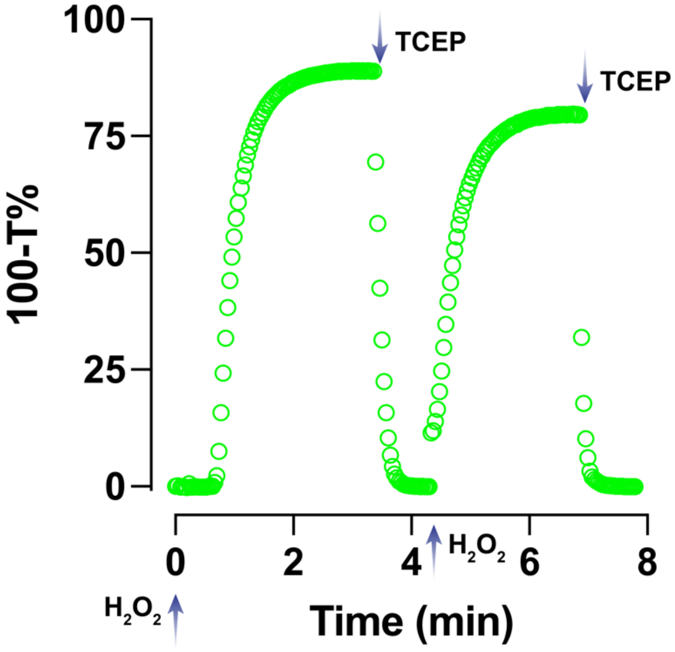


**Supplementary figure 6.** The alternating addition of $H_2O_2$ and TCEP show condensation and dissolution of NADPH/DGR$_5$Cs-sCR$_5$GD coacervate droplets. The mixtures were prepared in PBS buffer, at the concentration of $NADP^+$ 4 mM, DGR$_5$Cs-sCR$_5$GD 20 mM (monomers basis), $H_2O_2$ 2.5 mM, and TCEP 0.5 mM.

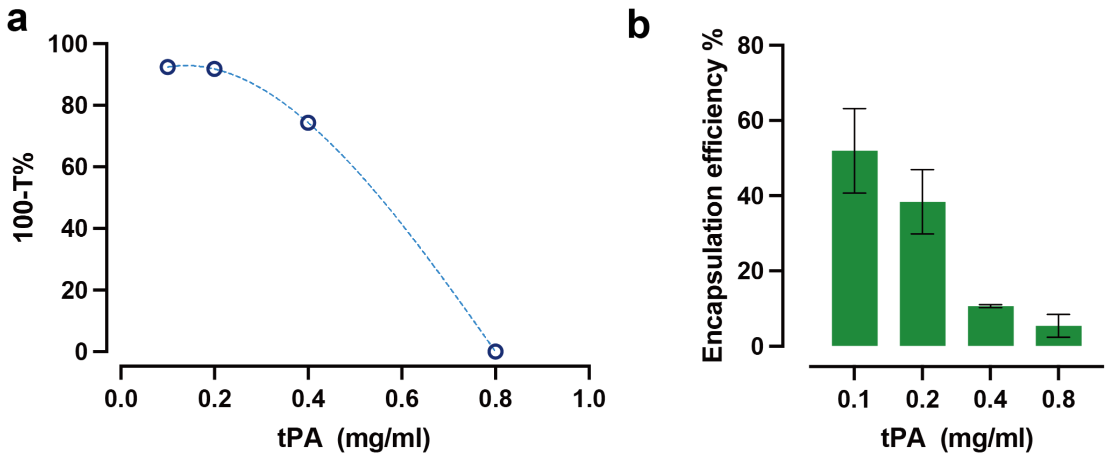


**Supplementary figure 7.** (a) Turbidity of NADPH/$R_{10}$ coacervates as a function of tPA concentration. (b) Encapsulation efficiency of tPA with increasing tPA concentration. All error bars represent standard deviation. (n=3).

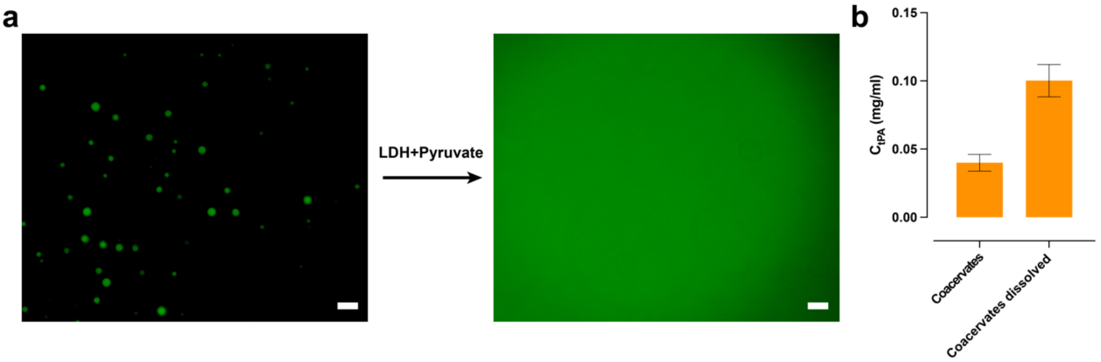


**Supplementary figure 8.** (a) Fluorescence microscopy images of NADPH/DG$R_5$Cs-sC$R_5$GD coacervates (left), and after addition of LDH-pyruvate mixture (right), showing release of FITC-tPA. Scale bars, 20 μm. (b) The tPA concentration in the supernatant was measured both before and after the dissolution of the NADPH/DG$R_5$Cs-sC$R_5$GD coacervates through LDH-pyruvate mixture addition. All error bars represent standard deviation. (n=3).

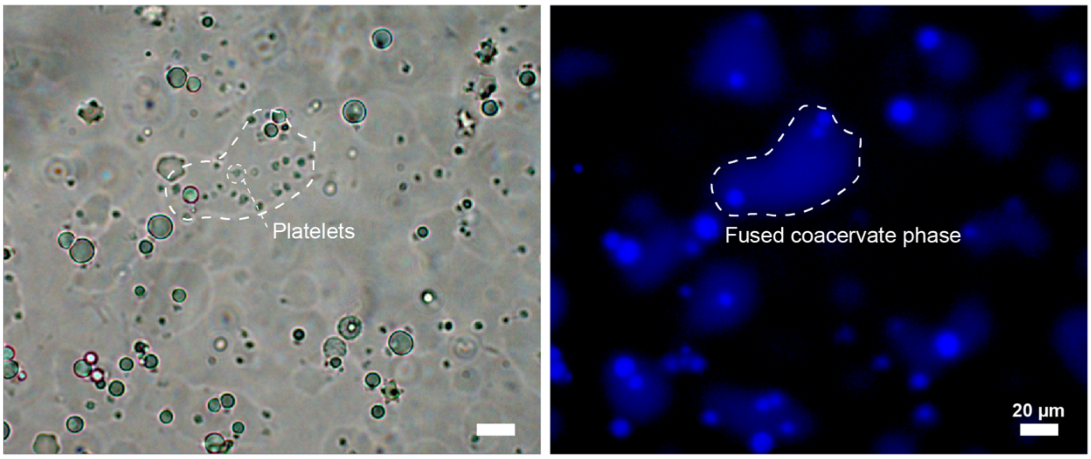


**Supplementary figure 9.** Bright-field and fluorescence images of NADPH/R*pep*-RGD coacervate droplets incubated with activated platelets. After the coalescence of the droplets on the slide, we observed the activated platelets concentrated on the fused coacervate phase. Scale bar, 20 μm.

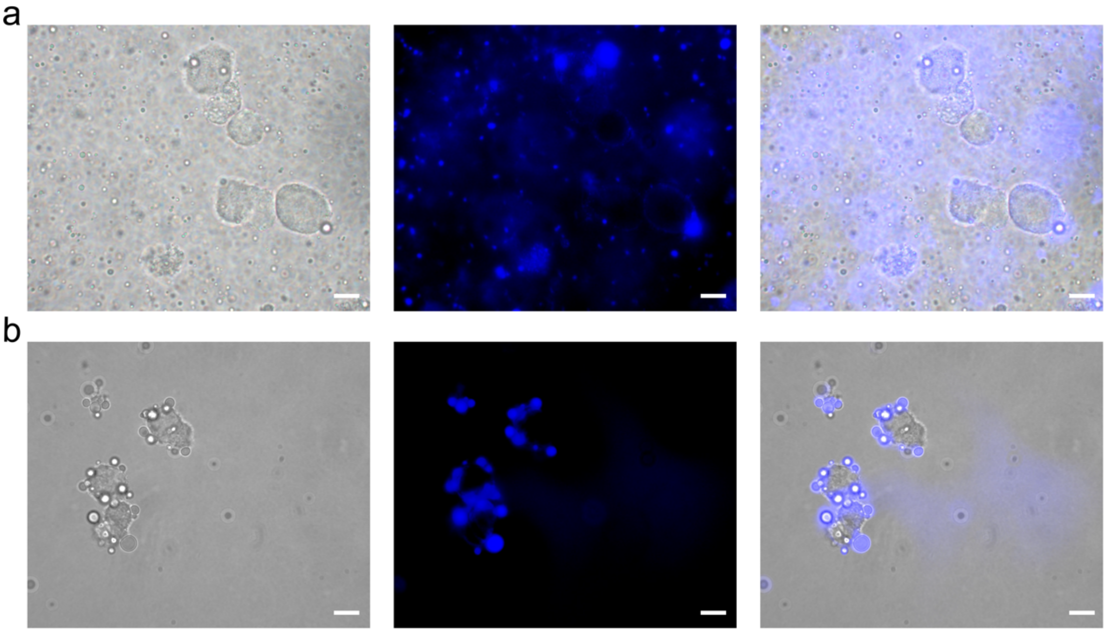


**Supplementary figure 10.** (a) Brightfield (left), fluorescence (middle), and overlay (right) images of Hela cells interacted with FITC-tPA loaded NADPH/$R_{10}$ coacervates. (b) Brightfield (left), fluorescence (middle), and overlay (right) images of Hela cells interacted with FITC-tPA loaded NADPH/$R_{10}$ coacervates. Scale bar, 10 μm.

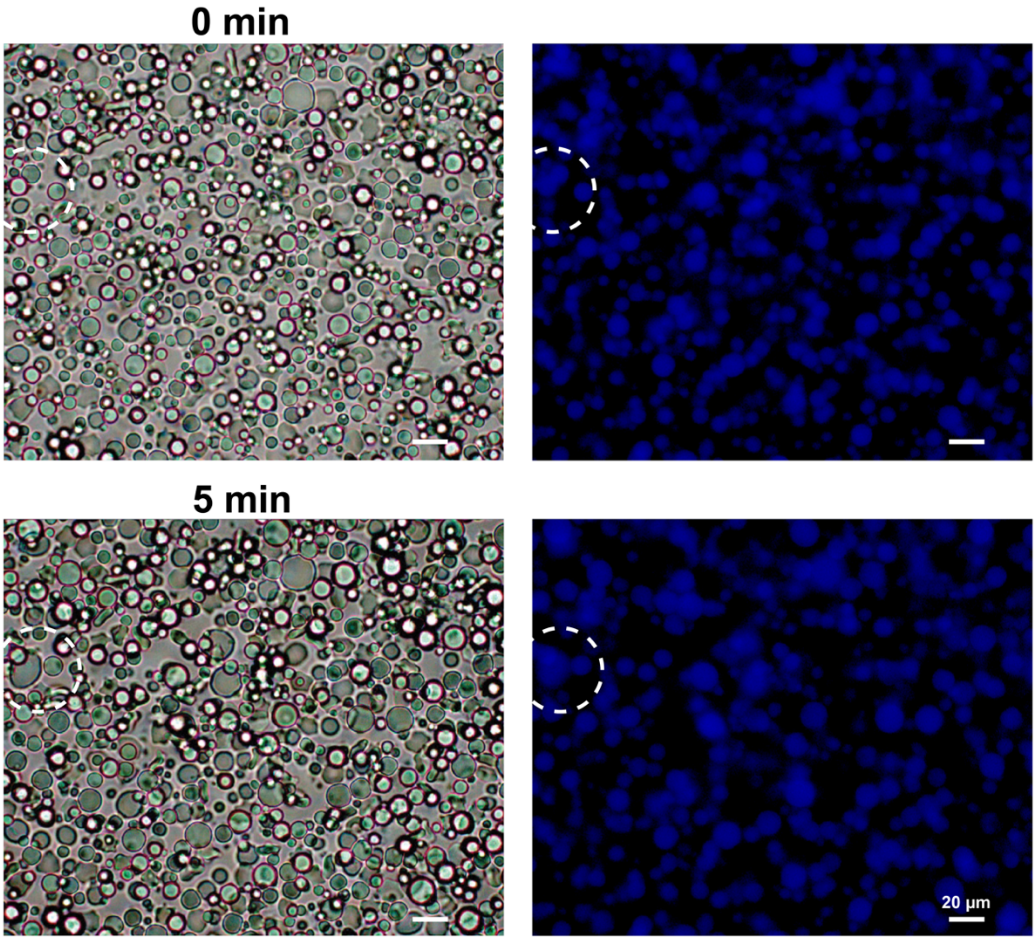


**Supplementary figure 11.** Slowly fusion of NADPH/R*pep*-RGD coacervate droplets (4 mM NADPH, 20 mM R*pep*-RGD (Arg monomer basis), in PBS buffer incubated with activated platelets). Scale bar, 20 µm.

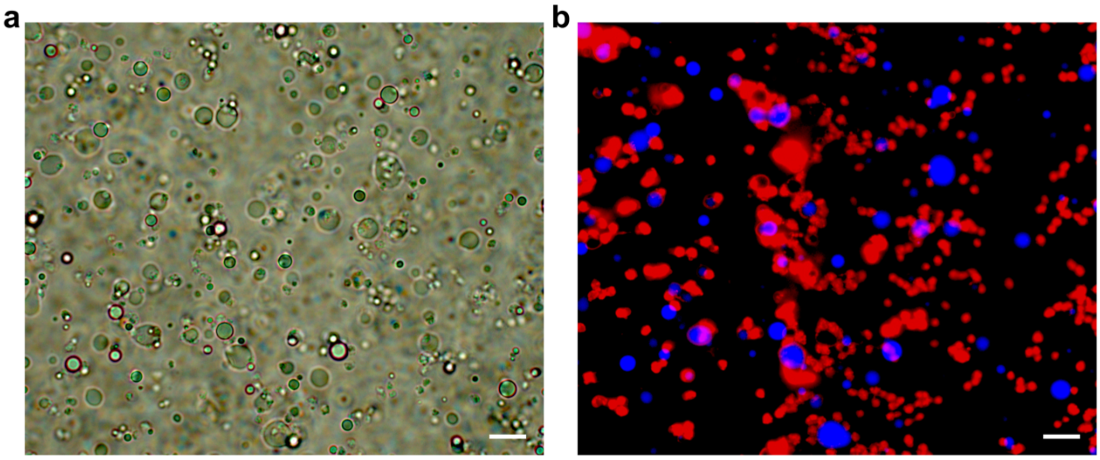


**Supplementary figure 12.** Brightfield (left), and fluorescence (right) images of Dil (red fluorescence)-stained platelets interacted with NADPH/$R_{10}$ coacervates. Scale bar, 20 µm.
