# Supplementary material for: Redox-responsive peptide-based complex coacervates as delivery vehicles with controlled release of proteinous drugs
Source: Commun Chem. 2023 Nov 7;6:243. doi: 10.1038/s42004-023-01044-8 (PMC10630460; doi:10.1038/s42004-023-01044-8)
Supplement: Supplementary file 3 — Description of Additional Supplementary Files [file 42004_2023_1044_MOESM3_ESM.pdf]

Description of Additional Supplementary Files

File name: Supplementary Data 1

Description: Source data points for Fig. 2cd

File name: Supplementary Data 2

Description: Source data points for Fig. 3b

File name: Supplementary Data 3

Description: Source data points for Fig. 3d

File name: Supplementary Data 4

Description: Source data points for Fig. 4ef

File name: Supplementary Data 5

Description: Source data points for Fig. 5c
